# Assessment of demographics factors and clinical staging in patients submitted to salvage surgery for oropharyngeal squamous cell carcinoma

**DOI:** 10.1016/S1808-8694(15)30599-1

**Published:** 2015-10-18

**Authors:** Helma Maria Chedid, Sérgio Altino Franzi

**Affiliations:** 1 ^1^ Master's degree student, graduation course in health sciences, Heliopolis hospital - Hosphel. Assistant in the Otorhinolaryngology and Head & Neck Surgery Department, Heliopolis hospital.; 2 Doctoral thesis in health sciences, Sao Paulo university; faculty member of the graduation course on health sciences, Heliopolis hospital - Hosphel. Assistant in the Otorhinolaryngology and Head & Neck Surgery Department, Heliopolis hospital. Heliópolis Hospital - Head & Neck Surgery.

**Keywords:** squamous cell carcinoma, salvage surgery, oropharynx

## Abstract

The usual management of upper aero digestive tract squamous cell carcinoma is surgery associated or not to post surgical radiotherapy. Loco-regional relapses constitute the main failure of the initial treatment and early diagnosis justifies the indication of salvage surgery.

**Aim:**

Descriptive analysis of demographic data and staging for salvage surgery of oropharynx tumors.

**Material and methods:**

We studied retrospectively 78 patients submitted to surgery in all cases; however, just 37 patients received post surgical radiotherapy.

**Results:**

There was a predominance of males in 70 cases, with mean age of 54.2 years, and 54 patients were Caucasian. The patients were classified as T3 and T4 in 38 cases and 40 patients were classified as N0 neck. 35 patients developed loco-regional distant relapses. 17 patients were submitted to salvage surgery and 12 patients were reclassified as T1; 2 patients T2 and in relation to the clinical stage N, 2 patients were N2a and 2 patients N2b. The average age of the patients submitted to salvage surgery was 52.8 years, with predominance of male Caucasians.

**Conclusion:**

Clinical stage I and II were accorded salvage surgery.

## INTRODUCTION

Surgery, with or with no postoperative radiotherapy, is the classical treatment of epidermoid carcinomas of the head and neck.^1^ Head and neck malignancies, especially the upper aerodigestive epidermoid carcinoma (UADEC), generally progresses with locoregional recurrence.

Locoregional recurrences are the most frequent cause of failure in the initial treatment of epidermoid carcinoma patients, particularly in advanced cases in which the diagnosis was made late.^2^

Local recurrence is common, occurring in 40% to 50% of patients treated initially with intentionally curative surgery, especially in advanced clinical stages of the disease.^3^ Such recurrences may be due to failure in the initial planning of therapy, to advanced clinical stages upon presentation, to the degree of molecular phenotype involvement, or to more aggressive tumor behavior. Most of these cases occur within the first 24 months of post-therapy follow-up.

Regional recurrences are treated more successfully due to a surgical salvage paradigm in clinically negative necks upon the initial presentation, and that were not subjected to elective neck dissection. It is a consensus that the success rate of salvage surgery of the neck after prior neck dissection is poor.^4^, ^5^, ^6^, ^7^

Salvage surgery, even in potential cases for this form of treatment, is controversial; most patients have a second recurrence or persistence of the disease within six months of salvage therapy.^8^ Physicians should take into account the morbidity, and mainly an unfavorable prognosis, when choosing the ideal treatment. In certain situations, the ideal treatment may be palliative support, even if the are potentially respectable lesions.

The clinical presentation of locoregional recurrence depends on the site of the primary tumor; indices vary from 25% to 48%. Gilbert and Kagan^2^ reviewed a number of series and showed that tonsillary and soft palate tumors tend to recur locally in a 2:1 frequency ratio compared with regional recurrences. The most frequent recurrence site for base of the tongue tumors is the endolarynx, by posterior extension of the tumor. Posterior wall tumors tend to recur locally after surgical treatment, in a 1:3 ratio compared with regional recurrences.

Evidence of single distance metastases is sparse. This may be an argument in favor of more aggressive “radical” therapy, namely major resection and reconstruction if there is local recurrence. Procedures include total glossolaryngectomy and pectoralis major muscle myocutaneous flap reconstruction. Regardless of the extension of surgery for the treatment of recurrences, the literature shows that the result of salvage procedures depends on the size of the initial tumor and the time for diagnosing recurrences. In other words, whether tumors were in initial clinical stages (I and II) and whether recurrences were detected one year following therapy. Selected cases of advanced tumors (clinical stages III and IV) upon the initial presentation, with early recurrences, may benefit from a second surgical procedure; these patients may progress favorably.^9^, ^10^

Other treatment modalities in locoregional recurrences are radiotherapy alone or with chemotherapy. It is reserved for non-resectable tumors and/or in patients unable to undergo surgery. Radiotherapy salvage depends on technical limitations concerning prior irradiation doses and the toxicity of chemotherapy, such as kidney failure and medullary suppression.^11^

Many medical variables thus influence decision-making in the locoregional recurrence of head and neck epidermoid carcinoma. These include the patient's age, his or her performance status, initial clinical staging and staging of locoregional recurrences, and the type of treatment.

The purpose of this paper was to make a descriptive analysis of demographic, clinical staging and progression data on locoregional recurrences of oropharyngeal tumors undergoing salvage surgery.

## SERIES AND METHODS

A retrospective study of 78 patients with histopathologically demonstrable oropharyngeal epidermoid carcinoma, treated initially by surgery with or with no postoperative radiotherapy, was carried out.

We defined locoregional recurrence in the oropharynx as tumors diagnosed no later than 24 months after treatment. A tumor arising in the same anatomical site 25 months or more after treatment was considered as a second tumor.

Demography (age, sex and racial group) and clinical staging data (tumor size and neck lymph nodes) were assessed upon admission of patients and when the diagnosis of locoregional recurrence was made. These findings, as well as the progression after therapy, were evaluated in patients with locoregional recurrences that underwent salvage surgery. The type of locoregional recurrence was also defined as exclusively local, exclusively regional, or locoregional; the distribution of salvage surgery according to the type of recurrence and the procedure done as a result was also noted.

The Research Ethics Committee of the Heliopolis Hospital approved this study (number 497) on 10 October de 2006.

## RESULTS

The mean follow-up time in our sample was 29.6 months. The mean time for diagnosing locoregional recurrences as of the initial treatment was 9.2 months; three cases were diagnosed more than 24 months after surgery.

In 78 patients, manifestations of a “new tumor” were as follows: 21 patients (27%) with local recurrences; 5 patients (6.4%) with regional recurrences; 8 patients (10.2%) with locoregional recurrences; 1 patient (1.3%) with local recurrences and distance metastases on separate dates; 2 patients (2.6%) with regional recurrences and distance metastases, which in one case were diagnosed in separate dates; 1 patient (1.3%) with regional recurrence and a second tumor in separate dates; 1 patient (1.3%) with a distance metastasis; and 7 patients (9%) with a second primary tumor. Absolute numbers for the “new tumor” manifestation type were: 38 patients (48.7%) with locoregional recurrences; 4 patients (5.1%) with distance metastases; and 9 patients (11.5%) with second tumors. Of 38 local and regional recurrences, local recurrences were found in 22 patients (58%), regional recurrences were found in 8 patients (21%), and locoregional recurrences were found in 8 patients (21%) (Table 1).

**Table 1:** Distribution of the type of locoregional recurrence.

Variable	Category	n	%
Recurrence	Local	22	58
	Regional	8	21
	Loco-regional	8	21

n= 38 patients.

Thirty-seven patients presented locoregional recurrences (a case in which a simultaneous diagnosis was made of a regional recurrence and a distance metastasis), of which 17 patients (45.9%) underwent salvage surgery and five patients were also treated with postoperative radiotherapy. Recurrence in 13 (76.5%) of these 17 patients was on the primary tumor site; recurrence in 4 (23.5%) of these 17 patients had neck recurrences. No patient with any locoregional recurrences underwent salvage surgery.

Thirteen (59%) of 22 patients with single local recurrences underwent salvage surgery; four (57.1%) of seven patients with single regional recurrences underwent salvage neck dissection.

There were 16 male patients (94.1%) and one female patient (5.9%) that underwent salvage surgery. Twelve patients (70.58%) were white, 4 patients (23.52%) were of mixed skin color and 1 patient (5.9%) was black. The age when a diagnosis of locoregional recurrence was made ranged from 24 to 69 years (mean 52.8 years). There was one patient (5.9%) aged between 21 and 40 years, 11 patients (64.7%) aged between 41 and 60 years, and 5 patients (29.4%) aged over 61 years.

Clinical staging in locoregional recurrence showed that 12 (92.3%) of 13 patients undergoing salvage surgery of the primary tumor were staged as T1 and only one patient (7.7%) was T2. Two (50%) of four patients undergoing salvage neck dissection were staged as N2b, and the other two (50%) were staged as N2c.

Clinical staging upon admission, before the initial treatment, of patients undergoing salvage surgery revealed that four patients (23.5%) were T1, seven patients (41.1%) were T2, five patients (29.5%) were T3, and one patient (5.9) was T4. Positive lymph nodes (N) were found as follows: 10 patients (58.8%) were N0, three patients (17.6%) were N1, one patient (5.9%) was N2a, one patient (5.9%) was N2b, and two patients (11.7%) were N2c.

Table 2 shows the results of salvage surgery types for the primary tumor and the neck.

**Table 2:** Distribution of salvage surgery according to the primary tumor and the neck

Variable	Category	n	%
Primary tumor	Tumor resection by a natural access route	10	77,0
	Partial glossectomy	2	15,3
*Neck	Buccopharyngectomy	1	7,7
	RND/MRND	4	67,0
	PND	2	33,0

* RND/MRND - radical / modified radical neck dissection. PND - posterior neck dissection.

A second recurrence was seen in 11 (64.7) of 17 patients; of these, six patients (54.5%) underwent a second salvage procedure, three patients (50%) due to local recurrences and three patients (50%) due to regional recurrences.

Only three (8.2%) of the remaining patients with locoregional recurrences underwent salvage radiotherapy. The other 17 patients (45.9%) were treated with palliative support measures.

The distance metastases sites were the subcutaneous neck tissues in three patients (75%) and the base of the cranium in one patient (25%). Locoregional disease control was achieved in three (75%) of these four patients.

A second oropharyngeal tumor was seen in three patients (33.4%); the soft palate was involved in two of these patients (66.7%) and the tonsillary region was involved in one patient (33.3%). There were six second tumor patients, as follows: one (11.1%) in the hard palate; one (11.1%) in the nasal fossa; one (11.1%) in the stomach; one (11.1%) is the distal esophagus; and two (22.2%) in the lungs. There were four 4 (44.4%) second tumors in the head and neck; three patients (75%) were treated surgically and one patient (25%) was treated with radiotherapy only. There were three cases of second primary tumors in the oropharynx, one of which had locoregional recurrence and was treated by salvage surgery.

The progression of the 17 patients (45.9%) that underwent salvage surgery until the last follow-up visit in the study was as follows: 10 patients (58.8%) were alive and disease-free after a minimum 13-month follow-up; 6 patients (35.3%) died due to locoregional disease; and one patient was alive but with regional disease and distance metastases.

## DISCUSSION

When diagnosing a locoregional recurrence that is resectable, therapy is not necessarily surgical. Other factors have to be taken into account before salvage surgery, such as what was the initial treatment and what is the extension of recurrence. Surgery certainly is the treatment of choice in cases of an early stage (I and II) locoregional recurrence, regardless of the first treatment. Detection of resectable lesions at advanced stages often means that reconstruction is necessary. In this situation, quality of life issues after salvage surgery should be taken into account, since there may be postoperative complications due to fibrosis from previous procedures or from radiotherapy, meaning that there is less control of the disease. Our results are similar to those found in the literature; salvage surgery was done in all early stage (I and II) local recurrences, regardless of the extension of resection in the initial treatment. Salvage surgery was also done in all cases of locoregional recurrences at early clinical stages.^6^, ^12^, ^13^

One should bear in mind that after three years of follow-up, the probability of a second tumor developing is greater than the possibility of local recurrence. A second tumor may develop on the site of a primary tumor that was removed surgically. It is thus difficult to establish whether a new tumor that develops on the healing site of a previously removed tumor is the result of field cancerization or the presence of residual disease cell niches.^14^ Although it may be difficult to define whether it is a local recurrence or a second tumor on the healing site of a previously removed cancer, salvage surgery of a second tumor diagnosed more than 24 months after an original lesion was removed has a better prognosis, implying in different results of therapy in these cases. There were three cases initially diagnosed as “new tumors,” of which two were eventually considered locoregional recurrences; these were not candidates for salvage surgery. The third case was defined as a second primary oropharyngeal tumor, and the patient underwent surgery and locoregional control of the disease within a 63.6-month follow-up period.

The rate of cervical cancer control following salvage surgery is higher in regional recurrence when palpable lymph nodes were absent upon the initial presentation or in patients not undergoing elective neck dissection. Our results are in line with these findings, given that patients undergoing salvage neck dissection had not been considered for neck dissection during the initial presentation. A further possibility is that such regional recurrences occurred out of previously manipulated areas, which suggests an increased success rate of salvage procedures.

An indication for contralateral neck dissection obeys the same oncological principles as those used when deciding for neck dissection ipsilateral to the primary tumor. The importance of indicating contralateral neck dissection in the initial presentation of this disease is based on contralateral regional recurrence rates. Kowalski et al.^15^ analyzed bilateral neck dissections in epidermoid carcinomas cases and showed that neck dissection should be done routinely in patients at a high risk of developing contralateral metastases. This author's follow-up revealed that 50% of patients with contralateral regional recurrences were not candidates for salvage surgery. Our study showed similar results, as only 50% of regional recurrences fulfilled the criteria for salvage neck dissection.

Amar et al.16 demonstrated statistically significant results in a study of contralateral cervical recurrence in epidermoid carcinomas of the mouth and larynx, showing that contralateral recurrence is related to the site and size of the primary tumor and the presence and number of ipsilateral lymph node involvement. This paper, however, found no relation between contralateral regional recurrence and the site of the primary tumor or lymph node involvement ipsilateral to the primary tumor.

Distance metastases are rare in oropharyngeal epidermoid carcinomas. A study of 1,244 head and neck epidermoid carcinoma patients revealed that 5% of the distance metastases presented with locoregional control of the disease. The diagnosis of distance metastases in 75% of our cases was made with locoregional control of the disease. Distance metastases developed concomitantly with local recurrence in only in only 25% of cases.^17^ Yonemoto et al.^18^ demonstrated that distance metastases developed in parallel with local recurrences. A diagnosis of distance metastases in the present study was made in 5.1% of cases during the follow-up period; one case (25%) was diagnosed upon locoregional control of the disease.

## CONCLUSION

Patients diagnosed with epidermoid carcinoma of the oropharynx and with locoregional recurrence, which underwent salvage surgery in this study, initially had predominantly early clinical staging (I and II); locoregional disease control was achieved in 58.8% of cases at a minimum 12 months after salvage.

## References

[1] Amar A, Curioni OA, Franzi SA, Rapoport A (2005). Recidivas locais após o tratamento cirúrgico do carcinoma epidermóide de cabeça e pescoço em estádio avançado. Rev Col Bras Cir.

[2] Gilbert H, Kagan AR (1974). Recurrence patterns in squamous cell carcinoma of the oral cavity, pharynx, larynx. J Surg Oncol.

[3] Kowalski LP, Agra GMI, Carvalho AL, Ulbrich FS, Carvalho OD, Magrin J (2006). Prognostic factors in salvage surgery for recurrent oral and oropharyngeal cancer. Head Neck.

[4] Goodwin W (2000). Salvage surgery for patients with recurrent squamous cell carcinoma of the upper aerodigestive tract: when do the ends justify the means. Laryngoscope.

[5] Wong LW, Wei WI, Lam LK, Yuen APW (2003). Salvage of recurrent head and neck squamous cell carcinoma after primary curative surgery. Head Neck.

[6] Gleich LL, Ryzenmam J, Gluckman JL, Wilson KM, Barret WL (2004). Recurrent advanced (T3 or T4) head and neck squamous cell carcinoma: is salvage possible?. Arch Otolaryngol Head Neck Surg.

[7] Amar A, Franzi SA, Rapoport A (2003). Evolution of patients with squamous cell carcinoma of upper aerodigestive tract. São Paulo Med J.

[8] Stell PM (1991). Time to recurrence of squamous cell carcinoma of the head and neck. Head Neck.

[9] Schwartz GJ, Mehta RH, Wenig BL (2000). Salvage treatment for recurrent squamous cell carcinoma of the oral cavity. Head Neck.

[10] Kokal WA, Neifeld JP, Eisert ER, Terz JJ, Laurence W (1983). Management of locoregional recurrent oropharyngeal carcinoma. Am J Surg.

[11] Lavertu P, Adelstein DJ, Saxton JP (1999). Agressive cocncurrente chemoradiotherapy for squamous cell head and neck cancer. Arch Otolaryngol Head Neck Surg.

[12] Pittam MB, Thornton H, Palmer BV, Chapman P, Shaw HJ (1982). Results and prognostic factors in salvage surgery for squamous carcinoma of the tongue. Br J Surg.

[13] Eckardt A, Barth EL, Kokemueller H, Wegener G (2004). Recurrent carcinoma of the head and neck: treatment strategies and survival analysis in a 20-years period. Oral Oncol.

[14] Slaughter DP, Southwick HW, Smejkal W (1953). Field cancerization in oral stratified squamous epithelium. Cancer.

[15] Kowalski LP, Bagietto R, Lara JR, Santos RL, Silva J.R, Magrin J. (2000). Prognostic significance of the distribution of neck node metastasis from oral carcinoma. Head Neck.

[17] León X, Quer M, Orus C, Venegas MDP, Lopez M (2000). Distant metastases in head and neck cancer patients who achieved loco-regional control. Head neck.

[18] Yonemoto RH, Ching PT, Byron RL, Riihimaki DU, Calif D (1972). The composite operation in cancer of the head and neck (commando procedure). Arch Surg.

